# NDD-ECHO: A standardised digital assessment tool to capture early life environmental and inflammatory factors for children with neurodevelopmental disorders

**DOI:** 10.1016/j.bbih.2025.101011

**Published:** 2025-05-08

**Authors:** Shrujna Patel, Velda X. Han, Brooke A. Keating, Hiroya Nishida, Shekeeb Mohammad, Hannah Jones, Russell C. Dale

**Affiliations:** a Kids Neuroscience Centre, The Children's Hospital at Westmead, Faculty of Medicine and Health, University of Sydney, NSW, Australia; b The Children's Hospital at Westmead Clinical School, Faculty of Medicine and Health, University of Sydney, Sydney, NSW, Australia

**Keywords:** Autism spectrum disorder, Tourette syndrome, Obsessive-compulsive disorder, Epigenetic, Exposome, Maternal immune activation

## Abstract

Neurodevelopmental disorders (NDDs), such as autism spectrum disorder (ASD), attention-deficit hyperactivity disorder (ADHD), obsessive-compulsive disorder (OCD), and Tourette syndrome are highly prevalent in childhood and are influenced by both genetic and environmental factors. The maternal immune activation (MIA) hypothesis suggests that inflammation during pregnancy can increase the risk of NDDs in offspring. However, current practice focuses mainly on capturing family history of neurodevelopmental and psychiatric disorders. Beyond this, capturing detailed early life environmental data in clinical settings remains challenging. To address this, we developed NDD-ECHO (Neurodevelopmental Disorders-Environmental and Clinical History Online), a standardised digital assessment tool designed to systematically collect maternal and child environmental histories, alongside neurodevelopmental symptom profiles. NDD-ECHO is designed to collaboratively collect information from caregivers with clinician input, covering family medical history, maternal pregnancy history and environmental exposures, child's environmental exposures (including infection) and child's diagnosis, clinical course, and function. The survey, built on the REDCap platform, was piloted in a cohort of 161 children with complex NDDs referred to a tertiary neurodevelopmental clinic. Our results demonstrate that nearly 80 % of children experienced loss of developmental skills, and many had symptom exacerbations triggered by infections or stress, supporting the role of environmental factors in NDD symptomatology. NDD-ECHO, available for free download via the REDCap Shared Library, has clinical and research applications, enabling standardised identification of environmental triggers and exploration of gene-environment interactions. This tool advances research by improving data collection on environmental risk factors and enhances personalised care for children with NDDs. Limitations include reliance on retrospective caregiver reports and the pilot cohort's specialised clinical setting, which may limit generalisability.

## 1 Introduction

Neurodevelopmental disorders (NDDs) such as autism spectrum disorder (ASD), attention deficit hyperactivity disorder (ADHD), Tourette syndrome (TS), and obsessive-compulsive disorder (OCD) affect ∼10 % of all children, and their prevalence is rising (Merikangas et al., 2010; Zablotsky et al., 2019; Zeidan et al., 2022). Neurodevelopmental and psychiatric disorders share common genetic aetiologies, with many studies describing the overlapping genetic architecture and related vulnerability genes across seemingly diverse brain disorders (Anttila et al., 2018; Schork et al., 2019; Romero et al., 2022). Indeed, family history of neurodevelopmental and psychiatric disorders is a known risk factor for NDDs in children (Sandin et al., 2014; Xie et al., 2019; Ghirardi et al., 2021). The majority of NDDs are attributed to common susceptibility loci, instead of rare gene variants (Anttila et al., 2018; Trifiletti et al., 2022). Many of the genes associated with autism risk are involved in synaptic formation, transcriptional regulation, and chromatin-remodelling pathways (De Rubeis et al., 2014).

It is increasingly recognised that the interplay between genetic and environmental factors contribute to the pathogenesis of childhood NDDs (Bale et al., 2010; Lipkin et al., 2023). Environmental influences on human brain development begin from preconception and continue until early adulthood (Tierney and Nelson, 2009). During early brain development, the central nervous system undergoes rapid and dynamic changes. Microglia, the immune cells of the brain, actively participate in refining neural networks through synaptic pruning (Paolicelli et al., 2011; Schafer et al., 2012) which is modulated by genetic factors and individual experience (Sekar et al., 2016; Neniskyte and Gross, 2017). Environmental exposures on the brain during critical windows of development can have broad effects on the developmental trajectory of the child, mediated by neuroinflammatory, metabolic, epigenetic, and endocrine mechanisms(Bale et al., 2010).

Many epidemiological studies provide evidence that common maternal inflammatory states during pregnancy are associated with increased risk of NDDs in offspring. These include maternal autoimmune diseases, obesity, gestational diabetes mellitus, pre-eclampsia, stress, depression, infection, exposure to toxins (pollution, smoking, alcohol), and low socioeconomic status (Han et al., 2021b). The maternal immune activation (MIA) hypothesis is supported by a large body of preclinical evidence demonstrating that immune insult/activation during pregnancy increases the risk of neurodevelopmental problems in offspring. However, the effects in humans are much more difficult to explore due to confounding variables, both known and unknown. Pre-existing maternal health problems, such as endometriosis and polycystic ovary syndrome (PCOS), use of assisted reproductive technology, and preterm birth have also been linked to increased risk of NDDs in offspring (Arpino et al., 2010; Liu et al., 2017; Chen et al., 2020). Many of these risk factors share genetic and environmental vulnerabilities; they are often related and co-occur, making it difficult to understand their individual and cumulative contributions to risk for NDDs. For example, a data linkage study showed that children exposed to multiple maternal inflammatory factors had a higher risk of ADHD, demonstrating a cumulative effect of maternal inflammation on risk of NDDs in offspring (Nielsen et al., 2021). Though data linkage and cohort studies increasingly support an association between early life inflammation and altered neurodevelopment, causality and disease mechanisms remain unproven.

Environmental exposures in the postnatal period, such as infections, stress, and trauma, which represent second ‘hits’, have been observed to trigger the onset or worsening of neurodevelopmental or psychiatric symptoms in children. Increased early childhood infections have been observed in children with existing NDDs (Atladóttir et al., 2010; Jyonouchi et al., 2014; Lin et al., 2017; Karlsson et al., 2022; Merzon et al., 2023; Zhang et al., 2023). Parents also commonly report a loss or decline of developmental skills in their children, often triggered by periods of infections or stress (Jyonouchi et al., 2008; Jones et al., 2021). Jyonouchi et al. proposed that there is a subset within the ASD population that have a distinctive clinical profile, marked by “frequent infections, accompanied by exacerbation of behavioural symptoms and/or loss in acquired skills”(Jyonouchi et al., 2008).

The growing evidence highlights the importance of capturing detailed data regarding maternal inflammation and early life environmental exposures, as well as neurodevelopmental symptom profiles to better understand risk factors and phenotypic subsets in NDDs. The increasing use of diagnostic investigations (genomics, other omics) in NDD and neurology clinics also necessitates standardised clinical phenotyping to enable meaningful interpretation of results. Beyond collecting family history of neurodevelopmental and neuropsychiatric disorders, it is not a standard practice in NDD clinics to assess a wide range of maternal and child environmental risk factors, even those with strong links to NDD risk in offspring. There is no standardised methodology for routine clinical data collection, making it difficult to compare cohorts and investigate environmental risk factors in real-world clinical cohorts. One of the reasons for this is the lack of a clinical instrument to capture this information in a feasible, standardised manner.

To address this problem, we designed a clinical assessment tool – called NDD-ECHO (Neurodevelopmental Disorders-Environmental and Clinical History Online). NDD-ECHO is a digital instrument (REDCap platform) that captures a wide range of early life environmental exposures and detailed clinical profile in children who have a suspected or confirmed neurodevelopmental disorder. The tool is designed to be completed predominantly by the primary caregiver, prior to their clinic appointment. Here, we outline the process of choosing and compiling relevant questions to construct NDD-ECHO and present a pilot study of embedding the tool in a tertiary referral neurodevelopment clinic at the Children's Hospital at Westmead (Sydney, Australia).

## 2 Methods

NDD-ECHO was built and hosted on the REDCap (Research Electronic Data Capture) platform, a secure web application for online data collection and management. We aimed to standardise clinical data collection by providing binary fields (yes/no, presence/absence), or discrete options (multiple choice, checkboxes) and eliminating free text fields where possible. The goal was to produce systematically collected and standardised datasets for both clinical and research purposes.

NDD-ECHO was designed as a collaborative assessment tool, with temporally spread input from the primary caregiver of the patient and the treating clinician. Certain sections were designed to be emailed to the primary caregiver to complete prior to the child's clinic appointment. Some other sections were designed to be delivered to the primary caregiver over the phone by a member of the research/clinical team, as they may require some explanation of medical terminology. The treating clinician can then review the information and complete any remaining sections during the clinic appointment.

**Fig. 1:**
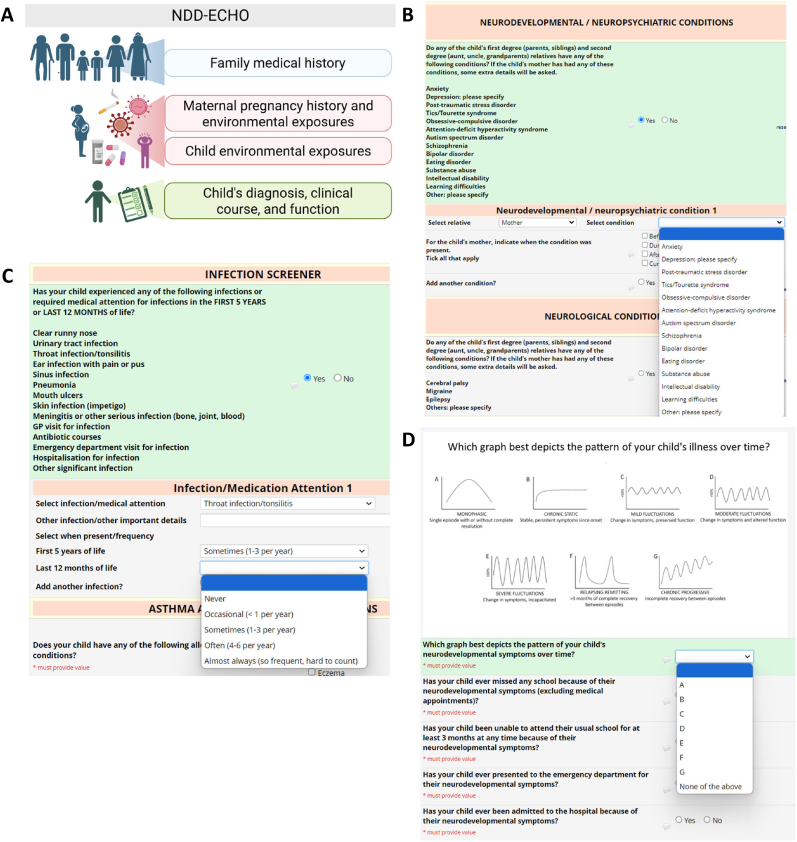
**The structure and layout of NDD-ECHO Survey on REDCap. A.** NDD-ECHO is a digital assessment tool, divided into four main sections: Family medical history, Maternal pregnancy history and environmental exposures, Child's environmental exposures (including infection), and Child's diagnosis, clinical course, and function. **B.** Family medical history is captured in NDD-ECHO using binary and multiple choice (dropdown) questions on REDCap. **C.** Our purpose-built infection screening tool captures common viral and bacterial infections in childhood using binary and multiple choice (dropdown) questions on REDCap. **D.** To evaluate the clinical course of the child's neurodevelopmental symptoms, parents were asked to select the figure most representative of the child's pattern of symptoms from seven different visual charts. Markers of disease severity are captured using binary questions on REDCap.

The tool takes 40–60 min to complete and is divided into four main sections (Fig. 1A). The scope of questions covered in these sections was derived from our previous work as well as evidence from literature as summarised in each section below.

1. Family medical history
2. Maternal pregnancy history and environmental exposures
3. Child's environmental exposures (including infection)
4. Child's diagnosis, clinical course, and function

### 2.1 NDD-ECHO section 1: Family medical history

The first section of NDD-ECHO is a comprehensive family history questionnaire to evaluate the overall medical and genetic background of the patient and family. We previously developed and piloted a paper-based family history questionnaire on 400 children and their families, with a focus on the first- and second-degree family history of autoimmunity and inflammatory states (Jones et al., 2021). Based on this work and our previous systematic review (Han et al., 2021c), NDD-ECHO was designed to capture detailed family medical history, with specific focus on environmental exposures that are known to increase the risk of NDDs in offspring. Since medical terminology may be unfamiliar to a lay audience, this section is designed to be delivered to the primary caregiver by a member of the clinical/research team over the phone, prior to their clinic appointment.

Firstly, we define the family tree, which includes the first-degree (biological parents and siblings) and second-degree (maternal and paternal grandparents, aunts, and uncles) relatives of the patient. The Survey then lists a range of medical conditions, grouped by categories, and then asks whether these conditions are present in any of the child's first- and second-degree relatives (Fig. 1B–Supp. Table 1). Since family history of NDDs is known to be a common risk factor, we first ascertain the history of neurodevelopmental and neuropsychiatric disorders in the family (Fig. 1B). We then collect family history of neurological conditions, autoimmune conditions, asthma, and allergic conditions. The final category includes any other medical problems in the family, including cardiovascular issues, metabolic disorders, and cancer. If any medical conditions are present in the child's mother, additional questions are asked, including the age and time of diagnosis (pre-pregnancy, during pregnancy, or postnatal) and whether exacerbations were experienced during pregnancy.

### 2.2 NDD-ECHO section 2: Maternal pregnancy history and environmental exposures

In addition to the information captured in Section 1, NDD-ECHO captures further details regarding maternal pregnancy history and environmental exposures that are known to increase the risk of NDDs in offspring (Supp. Table 2). This section is also designed to be delivered to the primary caregiver (usually the biological mother) by a member of the clinical/research team over the phone, prior to their clinic appointment. This section begins by capturing mode of conception (natural or assisted), followed by common pregnancy risk factors for NDD in offspring, including infections, pre-pregnancy body mass index, medication use, smoking, and alcohol consumption (Supp. Table 2). Low socioeconomic status is a known risk factors for NDDs and contributes to chronic systemic inflammation, thus it was included in NDD-ECHO (Furman et al., 2019). Socioeconomic status was assessed using Socio-Economic Indexes for Areas (SEIFA), which is a standardised socioeconomic score publicly available through the Australian Bureau of Statistics (Australian Bureau of Statistics, 2016). The mother's postal area code during pregnancy was used to generate the SEIFA score. Similar area code based socioeconomic scores are available in many other countries. While SEIFA provides standardised area-level SES measures, we note that future iterations of the tool should incorporate individual-level variables such as parental education or occupation to improve accuracy and international applicability. We selected the Pregnancy Complication Scale (Leckman et al., 1990) to characterise the overall physical difficulties/complications of the pregnancy. This scale was previously used and published by Leckman et al. in a study exploring perinatal risk factors in Tourette's syndrome (Leckman et al., 1990). It is used here as a brief and broad screening question, followed by a list of common perinatal complications, noted as present or absent. Finally, mothers are asked to rate their experience of life events during the pregnancy using the Level of Stress Severity question, also previously used by Leckman et al. (1990). (Supp. Table 2).

### 2.3 NDD-ECHO section 3: Child's environmental exposures

The next section was designed to capture the child's medical history and early life environmental exposures, mainly infection and stress. It is hypothesised that environmental factors interact with genetic vulnerabilities, possibly via epigenetic mechanisms (gene regulation, chromatin, transcription), and result in altered immune and brain function in children with complex NDDs (Han et al., 2021a). Therefore, capturing the presence of infectious disease, allergy, and autoimmunity (as a marker of vulnerability to immune dysregulation) is important in the proband. This section is also designed to be delivered to the primary caregiver by a member of the clinical/research team over the phone, prior to their clinic appointment.

The child's medical history is collected in a similar manner to Section 1 (Supp. Table 3). The presence of any additional medical conditions in addition to the neurodevelopmental/neuropsychiatric symptoms is noted, including neurological, autoimmune conditions, asthma, allergies, and any other medical problems. In clinical practice, children with NDDs often report recurrent infections in childhood, frequent use of antibiotics, and visits to the general practitioner for infections (Atladóttir et al., 2010; Jyonouchi et al., 2014; Lin et al., 2017; Karlsson et al., 2022; Merzon et al., 2023; Zhang et al., 2023). There are no formal definitions for recurrent infections in children and most infection assessment tools are focused on detecting immunodeficiencies. Therefore, we created a purpose-built infection screening tool (Fig. 1C–Supp. Table 3) for children, which captures common viral and bacterial infections in childhood (e.g. respiratory tract, urinary tract), as well as more serious bacterial infections (e.g. meningitis, bone infections). Rates of recurrent skin infections (impetigo) and mouth ulcers are also included, as previous work by our team has shown high rates of mouth ulcers in children with NDDs (Jones et al., 2021). Antibiotic courses, visits to general practitioners for infection treatment, emergency department visits, and hospitalisations for infections are also included. A 5-point Likert scale is used to assess frequency of infections: 0, Never; 1, Occasional (<1 per year); 2, Sometimes (1–3 per year); 3, Often (4–6 per year); 4, Almost always (so frequent, hard to count). Parents are asked to answer the infection screener with respect to the first five years of the child's life and the 12 months prior to the survey (Fig. 1C–Supp. Table 3). Exposure to stress and trauma is captured by asking if the child had experienced a stressful life event (e.g. loss of loved one), including the child's age when the event occurred and whether the child had a noticeable stressful reaction to the event (as determined by the caregiver).

#### 2.3.1 NDD-ECHO section 4: Child's diagnosis, clinical course, and function

The final component of NDD-ECHO includes documentation of the child's clinical diagnosis and functional status. This section has two components: 1) a battery of caregiver-reported questionnaires that are emailed to the primary caregiver to complete prior to their child's clinic appointment, and 2) a clinician-directed interview during the clinic appointment.

For the caregiver battery, we selected gold-standard, validated, free-to-use assessment tools to screen across broad neurodevelopmental domains. We aimed to embed these tools into clinical practice without adding cost and unreasonable burden on the family or the clinician. We included the Strengths and Difficulties Questionnaire (SDQ) to screen for broad emotional and behavioural problems (Goodman, 1997, 2001; Goodman and Goodman, 2009; Grasso et al., 2022), the Revised Child Anxiety and Depression Scale – Parent (RCADS-P) as a measure of mood and emotional symptoms (Chorpita et al., 2005; Ebesutani et al., 2010, 2015), the Affective Reactivity Index (ARI) as a short measure of irritability (Stringaris et al., 2012), the Conners Abbreviated Rating Scale to assess attention (Parker et al., 1996), and the EQ-5D-Y for general quality of life (Wille et al., 2010). We also included some bespoke questions (binary, checkbox, and multiple-choice), that were designed by our team to collect information about the clinical disease course that was not covered in the gold-standard tools. In clinical practice, it is observed that children with NDDs have different clinical courses in terms of neurodevelopmental symptoms. Some experience a monophasic infection-provoked deterioration, some have fluctuations (mild, moderate or severe), whereas some have more severe relapsing-remitting type of symptoms. To evaluate the clinical course of the child's neurodevelopmental symptoms, parents are asked to select the figure most representative of the child's pattern of symptoms from seven different visual charts (Fig. 1D). In addition, it is observed that children with NDDs often experience loss of previously acquired developmental skills, often termed developmental regression. Loss of skills, including loss of learning ability (attention/concentration), language, social skills, fine and gross motor skills are recorded in binary fashion (presence or absence). The presence of any known triggers (e.g., stress, infection, excitement) that exacerbate neurodevelopmental symptoms are also noted. Binary (yes/no) questions regarding prolonged periods of absence from school, emergency department visits, and hospital admissions are included as “markers of disease severity” Fig. 1D).

The clinician-directed interview during the clinic appointment is designed to fill any gaps in the previous sections, seek clarifications or additional information, and confirm the child's diagnosis and clinical plan. The child's diagnoses and comorbidities are determined using DSM-5 criteria (Regier et al., 2013). Symptom specific gold-standard assessment tools may be delivered by the treating clinician if deemed appropriate. These include the Children's Yale-Brown Obsessive Compulsive Scale (CY-BOCS) (Scahill et al., 1997; Storch et al., 2019; Kook et al., 2024), which is the most widely used measure of clinician-rated obsessive-compulsive symptom severity, and the Yale Global Tic Severity Scale (Leckman et al., 1989; Storch et al., 2005) if tics are present. The presence of ‘fear episodes’ with fight or flight symptoms is also recorded, along with their frequency and impact on the child's function.

We piloted NDD-ECHO in a new prospective clinical cohort of children aged <18 years with complex neurodevelopmental disorders referred to Prof Russell Dale's specialist clinic at the Children's Hospital at Westmead, Sydney, Australia from February 2021 to April 2024. In this clinic, there is a referral enrichment of Tourette syndrome/tic disorder and OCD, often with a phenotype of paediatric acute-onset neuropsychiatric syndrome (PANS) and autistic regression, with accompanying disorders such as ASD and ADHD. We present here a new clinical cohort of 161 children with complex NDDs who completed NDD-ECHO, to demonstrate the utility of this tool in an individual and cohort setting. We utilised data quality features in REDCap to minimise missing values, such as automated data checks and flagging of incomplete responses for follow-up with families when possible. The clinical team aimed for completeness of data during clinic appointments; however, some missing data was unavoidable. In this cohort, we have only reported definitive history and not employed any imputation strategies for missing data to avoid overcalling/overscoring. This tool was piloted in children aged 3–18 years and is intended for use in this age range. Section 4 assessments were selected based on validation in child and adolescent populations and were used in an age-appropriate manner. To highlight the value of the gold-standard free-to-use assessment tools, such as the SDQ, we present data from a control group of 58 age- and sex-matched children of hospital workers. These children did not have any NDDs, autoimmune diseases, or severe allergic conditions. Ethical approval was granted by the Sydney Children's Hospitals Network Human Research Ethics Committee (2021/ETH00356).

## 3 Results

### 3.1 Clinical characteristics of the NDD cohort

A cohort of 161 children with NDDs (mean age 10 years, range 3–18 years, 67 % males) were included (Table 1). After clinician DSM-5 assessments, the main neurodevelopmental diagnoses were tic disorder/Tourette syndrome (n = 116, 72 %), ADHD (n = 88, 55 %), OCD (n = 71, 44 %), and ASD (n = 58, 36 %). Anxiety disorder was common, present in 106 children (66 %). A group of 45 children (28 %) presented with a PANS phenotype, characterised by abrupt onset OCD and neuropsychiatric symptoms, often triggered by infection. Asthma and other allergic disorders were very common comorbidities, present in 97 children (60 %). The rate of autoimmune disease was also high, present in 18 children (11 %, Table 1).

**Table 1:** Clinical characteristics and comorbidities of the neurodevelopmental disorder patient cohort (n = 161).

	n (%)
**Age (mean (range))**	10 (3–18)
**Sex**	
Male	108 (67)
Female	51 (32)
Non-binary	2 (1)
**Neurodevelopmental/neuropsychiatric diagnoses**^a^	
Tic disorder/Tourette syndrome	116 (72)
Anxiety disorder	106 (66)
Attention-deficit hyperactivity disorder (ADHD)	88 (55)
Obsessive compulsive disorder (OCD)	71 (44)
Autism spectrum disorder (ASD)	58 (36)
Paediatric acute neuropsychiatric syndrome (PANS)	45 (28)
Emotional dysregulation NOS	26 (16)
Depression	19 (12)
Oppositional defiant disorder (ODD)	8 (5)
Post-traumatic stress disorder (PTSD)	6 (4)
Eating restriction/disorder	6 (4)
Functional neurological disorder (on NDD background)	5 (3)
Intellectual disability/global developmental delay (ID/GDD)	4 (2)
Learning disorder NOS	4 (2)
**Comorbidities**^a^	
Asthma and allergic disorders	97 (60)
Asthma	54 (34)
Allergy	42 (26)
Eczema	22 (14)
Hay fever	18 (11)
Autoimmune disease	18 (11)
Celiac disease	5 (3)
Psoriasis, Type 1 diabetes, Vitiligo, Other inflammatory arthritis (not specified)	2 (1)
Autoimmune cytopenia, Autoimmune encephalitis, Celiac disease, Graves' disease, Hashimoto's thyroiditis, Inflammatory bowel disease, Systemic lupus erythematosus, Thyroid disease not otherwise specified, Other autoimmune (not specified)	1 (1)
Hearing loss/impairment	4 (2)
Epilepsy	2 (1)

Abbreviations: NDD – neurodevelopmental disorder, NOS – not otherwise specified.

a Children with more than one diagnosis were included in the count for each individual diagnosis.

The clinical course of NDD symptoms varied; however, many children experienced severe (n = 56, 36 %) or moderate fluctuations (n = 51, 32 %). Approximately half of the children experienced a trigger at the onset of their neurodevelopmental symptoms (n = 77, 48 %, Table 2) and infection was the most common trigger (n = 44, 27 %), followed by stress (n = 24, 15 %). Most of the children (n = 141, 88 %) experienced exacerbations in neurodevelopmental symptoms throughout the clinical course, with the most common triggers being stress (n = 133, 83 %) and infection (n = 94, 58 %). One hundred and twenty-nine children (80 %) were reported to have a loss of one or more developmental skill domain at some stage during their history. A loss of learning ability (n = 93, 58 %) and social skills (n = 67, 42 %) were the most commonly reported. A smaller percentage (17–29 %) experienced other markers of severity (e.g. missed school, emergency department visit, and hospital admission). Almost half of the cohort had experienced a stressful life event (n = 72, 45 %). The majority of children were taking at least one medication (n = 119, 74 %), with the most common being clonidine (n = 38, 24 %), methylphenidate (n = 28, 17 %), fluoxetine (n = 26, 16 %), and aripiprazole (n = 25, 16 %, Table 2).

**Table 2:** Clinical course of neurodevelopmental symptoms and medications in the patient cohort (n = 161).

	n (%)
**Clinical course (see**Fig. 1D)^a^	
Severe fluctuations	58 (36)
Moderate fluctuations	51 (32)
Mild fluctuations	19 (12)
Chronic progressive	15 (9)
Chronic static	3 (2)
Relapsing remitting	6 (4)
Monophasic	2 (1)
**Trigger at onset of NDD symptoms**	77 (48)
Infection	44 (27)
Stressful event	24 (15)
Vaccination	4 (2)
Medication	3 (2)
Other	10 (6)
**Exacerbation of NDD symptoms at any time, triggered by**	141 (88)
Stress	133 (83)
Infection	94 (58)
Excitement	60 (37)
Fatigue	10 (6)
Other	3 (2)
**Loss of skills at the onset of NDD symptoms**	122 (76)
Learning ability (e.g. attention and concentration)	93 (58)
Social Skills	67 (42)
Fine motor skills (e.g. handwriting)	66 (41)
Gross motor skills (e.g. balance and coordination)	66 (41)
Language (loss of vocabulary)	43 (27)
Other	3 (2)
**Loss of skills at any time in the clinical course**	129 (80)
Learning ability (e.g. attention and concentration)	101 (63)
Social Skills	72 (45)
Fine motor skills (e.g. handwriting)	69 (43)
Gross motor skills (e.g. balance and coordination)	68 (42)
Language (loss of vocabulary)	44 (27)
Other	3 (2)
**Markers of severity (at any time in clinical course)**	
Presented to emergency department for NDD symptoms	47 (29)
Unable to attend school for >3 consecutive months	35 (22)
Admitted to hospital because of NDD symptoms	27 (17)
**Experienced stressful life event**	72 (45)
**Medications for neurodevelopmental symptoms or comorbidities**	
No medication	42 (26)
Any medication	119 (74)
Selective serotonin reuptake inhibitors (SSRIs) or tricyclic	
Fluoxetine	26 (16)
Fluvoxamine	10 (6)
Sertraline	6 (4)
Escitalopram	3 (2)
Clomipramine	2 (1)
Amitriptyline	1 (1)
Anti-psychotics	
Aripiprazole	25 (16)
Risperidone	14 (9)
Quetiapine	7 (4)
Paliperidone	1 (1)
Alpha-2 adrenergic agonists	
Clonidine	38 (24)
Guanfacine	28 (17)
Stimulants	
Methylphenidate	26 (16)
Dexamfetamine/lysdexamfetamine	13 (8)
Immune therapies	
Intravenous immunoglobulin	14 (9)
Azithromycin	11 (7)
Rituximab	1 (1)
Ruxolitinib	1 (1)
Other	
Cannabis	7 (4)
Atomoxetine	2 (1)
Lithium	2 (1)
Topiramate	1 (1)

Abbreviations: NDD – neurodevelopmental disorder.

a See Fig. 1D for the visual charts representing the seven different clinical courses presented in NDD-ECHO.

### 3.2 First-degree family medical history

History of neurodevelopmental and neuropsychiatric disorders was common in first-degree family members (n = 124, 77 %), particularly in mothers (n = 96, 60 %, Table 3). Anxiety and depression were the most common disorders in mothers and fathers. Tics/Tourette syndrome (n = 33, 20 %), OCD (n = 24, 15 %), ASD (n = 22, 14 %), and post-traumatic stress disorder (n = 22, 14) and were also commonly reported in first-degree relatives. A substantial group of mothers also had a history of autoimmune disorders (n = 39, 24 %) as well as asthma and allergic disorders (n = 89, 55 %, Table 3).

**Table 3:** Medical history of first-degree family members.^a^.

Family history	Any first degree relative n (%)	Mother n (%)	Father n (%)	Brother(s) n (%)	Sister(s) n (%)
**Neurodevelopmental and neuropsychiatric disorders**^b^	124 (77)	96 (60)	55 (34)	24 (15)	30 (19)
Anxiety	106 (66)	74 (46)	24 (15)	5 (3)	13 (8)
Depression	68 (42)	45 (28)	17 (11)	2 (1)	4 (2)
Attention-deficit hyperactivity syndrome	47 (29)	13 (8)	14 (9)	11 (7)	9 (6)
Tics/Tourette syndrome	33 (20)	7 (4)	11 (7)	9 (6)	6 (4)
Obsessive-compulsive disorder	24 (15)	10 (6)	5 (3)	0 (0)	9 (6)
Autism spectrum disorder	22 (14)	1 (1)	3 (2)	11 (7)	7 (4)
Post-traumatic stress disorder	22 (14)	16 (10)	4 (2)	1 (1)	1 (1)
Substance abuse	9 (6)	1 (1)	7 (4)	0 (0)	1 (1)
Bipolar disorder	6 (4)	3 (2)	3 (2)	0 (0)	0 (0)
Intellectual disability	4 (2)	2 (1)	0 (0)	0 (0)	2 (1)
PANS	4 (2)	0 (0)	0 (0)	1 (1)	3 (2)
Eating disorder	3 (2)	2 (1)	0 (0)	0 (0)	1 (1)
Learning difficulties	3 (2)	1 (1)	0 (0)	0 (0)	2 (1)
Borderline personality disorder	2 (1)	2 (1)	0 (0)	0 (0)	0 (0)
Schizophrenia	1 (1)	0 (0)	1 (1)	0 (0)	0 (0)
Other: please specify	1 (1)	0 (0)	1 (1)	0 (0)	0 (0)
**Neurology**^b^	47 (29)	39 (24)	10 (6)	4 (2)	9 (6)
Migraine	53 (33)	37 (23)	9 (6)	2 (1)	5 (3)
Cerebral palsy	4 (2)	1 (1)	0 (0)	2 (1)	1 (1)
Epilepsy	4 (2)	0 (0)	0 (0)	1 (1)	3 (2)
Others	2 (1)	2 (1)	1 (1)	0 (0)	0 (0)
**Autoimmune disorders**^b^	53 (33)	39 (24)	14 (9)	6 (4)	5 (3)
Psoriasis	12 (7)	6 (4)	6 (4)	0 (0)	0 (0)
Type 1 diabetes	7 (4)	1 (1)	2 (1)	3 (2)	1 (1)
Hashimoto's thyroiditis	7 (4)	7 (4)	0 (0)	0 (0)	0 (0)
Celiac disease	6 (4)	5 (3)	0 (0)	0 (0)	1 (1)
Crohn's disease	6 (4)	4 (2)	2 (1)	0 (0)	0 (0)
Thyroid disease not otherwise specified	6 (4)	4 (2)	0 (0)	1 (1)	1 (1)
Alopecia	5 (3)	1 (1)	3 (2)	1 (1)	0 (0)
Graves' disease	4 (2)	4 (2)	0 (0)	0 (0)	0 (0)
Ulcerative colitis	3 (2)	2 (1)	0 (0)	0 (0)	1 (1)
Vitiligo	3 (2)	2 (1)	1 (1)	0 (0)	0 (0)
Systemic lupus erythematosus	3 (2)	3 (2)	0 (0)	0 (0)	0 (0)
Rheumatoid arthritis	3 (2)	3 (2)	0 (0)	0 (0)	1 (1)
Multiple sclerosis/MOG disease	3 (2)	2 (1)	0 (0)	0 (0)	1 (1)
Ankylosing spondylitis	1 (1)	1 (1)	0 (0)	0 (0)	0 (0)
Bell's palsy	1 (1)	0 (0)	0 (0)	0 (0)	1 (1)
Juvenile idiopathic arthritis	1 (1)	0 (0)	0 (0)	1 (1)	0 (0)
Myasthenia gravis	1 (1)	0 (0)	1 (1)	0 (0)	0 (0)
Narcolepsy	1 (1)	1 (1)	0 (0)	0 (0)	0 (0)
Polymyalgia rheumatica	1 (1)	1 (1)	0 (0)	0 (0)	0 (0)
Sjogren's syndrome	1 (1)	1 (1)	0 (0)	0 (0)	0 (0)
Others	1 (1)	1 (1)	0 (0)	0 (0)	0 (0)
**Asthma and allergic disorders**^b^	120 (75)	89 (55)	51 (32)	25 (16)	38 (24)
Asthma	110 (68)	45 (28)	24 (15)	20 (12)	21 (13)
Hayfever	77 (48)	45 (28)	17 (11)	3 (2)	12 (7)
Allergy	63 (39)	30 (19)	13 (8)	9 (6)	11 (7)
Eczema	58 (36)	25 (16)	11 (7)	5 (3)	17 (11)
**Other medical conditions**^b^	96 (60)	78 (48)	36 (22)	3 (2)	6 (4)
High blood pressure	29 (18)	18 (11)	11 (7)	0 (0)	0 (0)
Endometriosis (mother/sister only)	20 (12)	19 (12)	0 (0)	0 (0)	1 (1)
Polycystic ovary syndrome (mother only)	20 (12)	19 (12)	0 (0)	0 (0)	1 (1)
High cholesterol	15 (9)	12 (7)	13 (8)	0 (0)	0 (0)
Cancer	12 (7)	9 (6)	4 (2)	0 (0)	1 (1)
Chronic fatigue	11 (7)	8 (5)	1 (1)	1 (1)	1 (1)
Type 2 diabetes	9 (6)	6 (4)	3 (2)	0 (0)	0 (0)
Cardiovascular disease	4 (2)	1 (1)	3 (2)	0 (0)	0 (0)
Periodontitis	3 (2)	2 (1)	1 (1)	0 (0)	0 (0)
Fatty liver disease	3 (2)	1 (1)	2 (1)	0 (0)	0 (0)
Fibromyalgia	1 (1)	4 (2)	0 (0)	0 (0)	0 (0)

Abbreviations: PANS – pediatric acute-onset neuropsychiatric syndrome.

a NDD-ECHO collected first- and second-degree family medical history, but we have presented only first-degree family history in the interest of simplicity.

b Family members with more than one diagnosis were only counted once the overall category totals but included separately in the count for each individual diagnosis.

### 3.3 Pregnancy history and complications

Most mothers reported natural conception (n = 143, 89 %, Table 4) and 34 (21 %) reported an infection during pregnancy. Approximately half of the mothers (n = 82, 51 %) had a pre-pregnancy body mass index in the healthy weight range. Rates of smoking (n = 13, 8 %) and alcohol consumption (n = 8, 5 %) during pregnancy were low. On the Pregnancy Complication Scale, 66 (41 %) of mothers reported a normal pregnancy, while 30 (19 %) reported mild or moderate complications, and 22 (14 %) mothers reported severe complications. High blood pressure (n = 18, 11 %) and gestational diabetes (n = 15, 9 %) were the most commonly reported pregnancy complications. On the Level of Stress Severity scale, 64 (40 %) of mothers experienced mild stress during pregnancy, while 18 (11 %) experienced moderate, and 21 (13 %) experienced severe stress (Table 4).

**Table 4:** Environmental exposures and pregnancy history of children in the neurodevelopmental disorder patient cohort

	n (%)
**Mode of conception**	
Natural	143 (89)
Assisted	13 (8)
**Infections during pregnancy**	34 (21)
**Pre-pregnancy body mass index (BMI)**	
Underweight (<18.50)	10 (6)
Healthy Weight Range (18.50–24.99)	82 (51)
Overweight (25.00–29.99)	28 (17)
Obese (>30)	25 (16)
**Smoking during pregnancy**	13 (8)
**Alcohol consumption during pregnancy**	8 (5)
**Pregnancy Complication Scale**^a^	
Optimal	7 (4)
Normal	66 (41)
Mild	30 (19)
Moderate	30 (19)
Severe	22 (14)
**Complications present during pregnancy or delivery**	85 (53)
High blood pressure	18 (11)
Gestational diabetes	15 (9)
Hyperemesis gravidarum	11 (7)
Pre-eclampsia	6 (4)
Spotting	6 (4)
Threatened preterm labour	6 (4)
Placenta praevia	3 (2)
Fetal abnormalities or antenatal scan	1 (1)
Group B Streptococcal colonisation	1 (1)
Others	18 (11)
**Level of Stress Severity**^a^	
None	42 (26)
Mild	64 (40)
Moderate	18 (11)
Severe	21 (13)
Extreme	10 (6)

a The Pregnancy Complication Scale and the Level of Stress Severity have been previously used and published by Leckman et al. in a study exploring perinatal risk factors in Tourette's syndrome. See Supp Table 2 for detailed definitions.

### 3.4 Early childhood infections

**Fig. 2:**
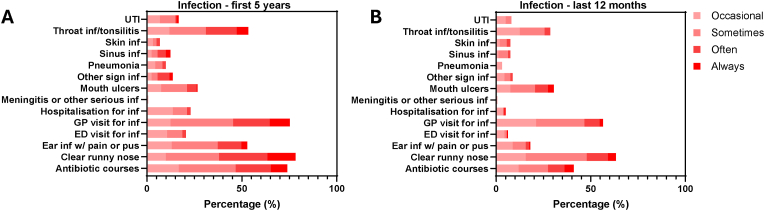
**Purpose-built infection screening tool captures early childhood infections**. **A**. Recurrent infections were common in the first five years of life, particularly clear runny nose, throat infections/tonsilitis, ear infections, and mouth ulcers. GP visits and antibiotic courses for infection were also frequently reported. **B**. Compared to the first five years of life, infections were less frequent in the 12 months of life prior to completing NDD-ECHO. Abbreviations: UTI – urinary tract infection, GP – general practitioner, ED – emergency department, inf – infection, sign – significant.

In the child, recurrent infections were common in the first five years of life, particularly clear runny nose, throat infections/tonsilitis, ear infections, and mouth ulcers (Fig. 2A). General practitioner visits and antibiotic courses for infection were also frequently reported (Fig. 2A). Compared to the first five years of life, infections were less frequent in the 12 months of life prior to completing NDD-ECHO(Fig. 2B).

### 3.5 Emotional and behavioural impairment using the Strengths and Difficulties Questionnaire (SDQ)

**Fig. 3:**
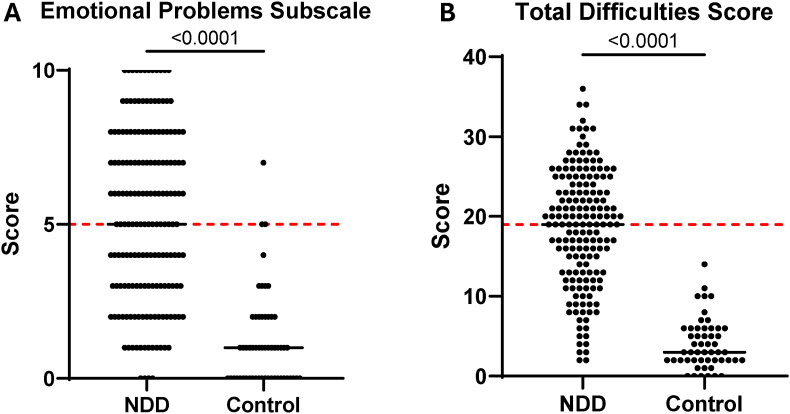
**Utility of the Strengths and Difficulties Questionnaire (SDQ).** The NDD cohort had significantly higher scores on the emotional problems subscale (p < 0.0001, **A**), as well as higher total difficulties scores (p < 0.0001, **B**) compared to the control group. Red dotted lines represent the cutoff for clinical concern – 90 % of children score below this cutoff according to Australian normative data for the SDQ (Mellor, 2005).

The NDD cohort were substantially impaired in terms of emotional and behavioural symptoms, as demonstrated by their increased scores on the SDQ compared to a control group of 58 age- and sex-matched children of hospital workers (mean age 9.5 years, range 5–14 years, 60 % males). The NDD cohort had significantly higher scores on the emotional problems subscale (p < 0.0001, Fig. 3A), as well as higher total difficulties scores (p < 0.0001, Fig. 3B) compared to the control group. Many children in the NDD cohort scored above the cutoff for clinical concern while almost all children in the control group scored below this cutoff (based on Australian normative data, represented by the red dotted lines in Fig. 3) (Mellor, 2005).

## 4 Discussion

Increasing evidence highlights the importance of capturing detailed data regarding maternal inflammation and early life environmental exposures to better understand risk factors and phenotypic subsets in NDDs. We initially designed a paper form to standardise data collection regarding environmental exposures in a NDD clinic setting and piloted this in a cohort of 200 children with tics and/or OCD (Jones et al., 2021). We have since revised this paper form and designed the NDD-ECHO on the REDCap platform to fill a critical gap in clinical practice and research. We now demonstrate the utility of NDD-ECHO in a second cohort, embedded in a tertiary referral NDD clinic setting.

In this cohort of 161 children, tic disorder/Tourette syndrome, ADHD, OCD, and ASD were the most common diagnoses. Since this clinic has referral enrichment for PANS, it was unsurprising that 45 children (28 %) presented with the PANS phenotype. Anxiety disorder was also present in 106 children (66 %), consistent with many previous studies showing high rates of mental health problems in children with NDDs (Simonoff et al., 2008; Hansen et al., 2018). Asthma, allergic conditions, and autoimmune diseases were commonly reported comorbidities in the children, consistent with previous literature indicating overlapping vulnerabilities between NDDs and immune-mediated conditions (Fasmer et al., 2011; Lyall et al., 2015; Hughes et al., 2018; Endres et al., 2022).

This study replicates our previous findings of high rates of neurodevelopmental/neuropsychiatric disorders and autoimmune disorders in first-degree family members, particularly mothers, of children with NDDs (Jones et al., 2021). While family history provides a clinical proxy for genetic vulnerability, it does not replace direct genetic measures such as searching for pathogenic DNA variants or polygenic risk scores. Future validation studies incorporating genomic data will be needed to strengthen the tool's capacity to explore gene-environment interactions. The purpose-built infection screener showed high rates of early childhood infections in this cohort, demonstrating a clinical manifestation of immune dysregulation. The infection screener represents our attempt to standardise data collection for this domain, addressing a current gap in the clinic, but we note it remains vulnerable to recall bias.

Nearly half of the cohort experienced a trigger (infection, stress) at symptom onset. Additionally, 88 % experienced exacerbations in symptoms triggered by stress or infection, consistent with our previous findings in this clinic (Jones et al., 2021). Together, these findings support the notion that environmental exposures, particularly infection, play a critical role in the clinical trajectory of children with NDDs and should be appropriately documented in the clinic.

Notably, 80 % of children experienced loss of one or more developmental skills (e.g., attention, motor, social, or language abilities) during their clinical history. This finding highlights the high prevalence of developmental regression among children with NDDs, consistent with previous findings from this clinic (Jones et al., 2019, 2021). According to these parental observations, neurodevelopment is not a ‘steady and consistent upward trajectory’, and setbacks are common. We note that this domain of regression is vulnerable to reporting effects and difficult to validate. However, frequent loss of acquired skills is a commonly reported concern of families in the context of NDDs, suggesting that developmental regression is a key feature in this population. Hence, it warrants data capture to enable closer clinical and research investigation to understand the triggers and patterns of these regressions.

Given the specialised nature of this clinic, it was unsurprising that the NDD cohort showed substantial impairment on the SDQ, demonstrating high levels of emotional and behavioural difficulties compared to the control group. We acknowledge that the highly selected control group (children of hospital workers) is likely to differ from the NDD cohort in many aspects (e.g. SES). The purpose of this control group was to simply demonstrate the impairment of the NDD group on the SDQ, hence we did not collect any additional information on the control group.

NDD-ECHO provides a standardised approach to capturing environmental and clinical data which has significant potential for improving personalised care in NDD clinics. To enable this standardised approach, we have made the Survey available for free download through the REDCap Shared Library. Routine use of the Survey could help clinicians identify key environmental risk factors and triggers, particularly triggers for developmental regression, contributing to better management and treatment of children with complex NDDs. For example, this tool could identify children with suspected infection-triggered symptom onset for targeted immunological assessment or flag cases with high maternal immune history for more personalised risk discussions. Standardising the collection of routine clinical data could also aid in advancing research on the gene-epigenetic-environment interplay in NDDs, especially in real-world clinical cohorts where detailed environmental histories are often lacking. The tool allows us to subgroup clinical cohorts to explore environment-disease associations. Pairing this clinical data with advanced biological investigations (e.g., peripheral blood immune phenotyping, transcriptomics, proteomics, and epigenetic assays) will enable translational research focusing on mechanisms of disease and therapeutics in NDDs. Future validation steps are required, including testing in broader clinical cohorts and community samples, linking with genetic data, and assessing predictive utility over time.

While NDD-ECHO captures comprehensive environmental and family history data, there are limitations in the self-reported nature of some sections. For example, recall bias regarding maternal pregnancy history or early childhood infections could influence the accuracy of the data. Incomplete data remains a limitation; however, it is unavoidable in a real-life clinical setting. This pilot was conducted in a specialised clinical setting, which may limit the generalisability of the findings to broader clinical practices. The cohort presented here is substantially impaired, as demonstrated by the markers of severity and high medication requirements (Table 2). Further studies using community and clinical cohorts that consider age, social, and seasonal variables are needed to better understand the ‘ranges of normality’ and therefore define meaningful differences in clinical cohorts across different domains (e.g. loss of skills, infection frequency). NDD-ECHO does require support by clinical research assistants to support completion of some sections and this added administrative burden may not be feasible in many clinical settings. Future efforts to revise this tool into a purely caregiver-reported format would be advantageous but may weaken the accuracy of the data due to limitations in understanding of medical terminology.

Future iterations of the tool should include paternal health information, especially in light of emerging evidence of paternal preconception factors (age-associated genetic and/or epigenetic factors). Addition of validated multi-item scales assessing chronic stress and childhood trauma should also be considered to better quantify these exposures. Physiological measures of stress (e.g. via wearables) could also be future area of research and integration with this tool.

NDD-ECHO successfully fills a critical gap by providing a feasible, standardised digital tool to capture detailed early life environmental influences in children with NDDs. Its implementation in clinical practice holds promise for enhancing the understanding of complex neurodevelopmental phenotypes and improving patient outcomes.

## CRediT authorship contribution statement

**Shrujna Patel:** Writing – review & editing, Writing – original draft, Visualization, Project administration, Methodology, Investigation, Formal analysis, Data curation, Conceptualization. **Velda X. Han:** Writing – review & editing, Methodology, Investigation, Data curation, Conceptualization. **Brooke A. Keating:** Writing – review & editing, Project administration, Methodology, Investigation, Data curation. **Hiroya Nishida:** Writing – review & editing, Project administration, Methodology, Investigation, Data curation. **Shekeeb Mohammad:** Writing – review & editing, Supervision, Methodology, Conceptualization. **Hannah Jones:** Writing – review & editing, Methodology, Investigation, Conceptualization. **Russell C. Dale:** Writing – review & editing, Supervision, Resources, Methodology, Investigation, Funding acquisition, Conceptualization.

## Funding

RCD is supported with an L1 investigator grant by the 10.13039/501100000925National Health and Medical Research Council (NHMRC) of Australia (ID 1193648) and the 10.13039/501100002337Petre Foundation.

## Declaration of competing interest

The authors declare that they have no known competing financial interests or personal relationships that could have appeared to influence the work reported in this paper.

## Data Availability

Data will be made available on request.
