# Supplementary material for: NDD-ECHO: A standardised digital assessment tool to capture early life environmental and inflammatory factors for children with neurodevelopmental disorders
Source: Brain Behav Immun Health. 2025 May 8;46:101011. doi: 10.1016/j.bbih.2025.101011 (PMC12140942; doi:10.1016/j.bbih.2025.101011)
Supplement: Multimedia component 1 [file mmc1.docx]

Supplementary Information: Table of Contents

**Supplementary Table 1. Capturing medical history of the child’s first- and second-degree relatives.**

**Supplementary Table 2: Capturing maternal pregnancy history and environmental exposures.**

**Supplementary Table 3. Capturing the child’s medical history and early life environmental exposures.**

**Supplementary Table 1. Capturing medical history of the child’s first- and second-degree relatives.** Section 1 of NDD-ECHO lists a range of medical conditions, grouped by categories, and captures the presence of these conditions in the child’s first- and second-degree relatives in a binary manner (presence/absence, see Figure 1B) on REDCap.

| **Category: Neurodevelopmental and neuropsychiatric conditions**  Anxiety  Depression  Post-traumatic stress disorder  Tics/Tourette syndrome  Obsessive-compulsive disorder  Attention-deficit hyperactivity syndrome  Autism spectrum disorder  Schizophrenia  Bipolar disorder  Eating disorder  Substance abuse  Intellectual disability  Learning difficulties  Other: please specify |
| --- |
| **Category: Neurological conditions**  Cerebral palsy  Migraine  Epilepsy  Others: please specify |
| **Category: Autoimmune conditions**  Hashimoto's thyroiditis  Graves' disease  Thyroid disease not otherwise specified  Type 1 diabetes  Vitiligo  Alopecia  Psoriasis  Celiac disease  Crohn's disease  Ulcerative colitis  Systemic lupus erythematosus  Lupus-like syndrome  Rheumatoid arthritis  Uveitis  Multiple sclerosis  Pernicious anemia  Autoimmune hepatitis  Polymyalgia rheumatica  Others: please specify |
| **Category: Asthma and allergic-type conditions**  Asthma  Allergy (e.g. grass, pollen, food allergies, medication allergies)  Hayfever  Eczema  Other inflammatory conditions: please specify |
| **Category: Other medical conditions**  High cholesterol  High blood pressure  Type 2 diabetes  Cardiovascular disease (e.g. Heart attack, angina, bypass surgery)  Polycystic ovary syndrome  Endometriosis  Fatty liver disease  Periodontitis  Cholestasis  Cancer: specify type of cancer  Chronic fatigue  Fibromyalgia  Others: please specify |

**Supplementary Table 2: Capturing maternal pregnancy history and environmental exposures.** Section 2 of NDD-ECHO captures further details regarding maternal pregnancy history and environmental exposures in using binary, checkbox, and multiple-choice questions on REDCap.

| **Mode of conception (natural or assisted)** |
| --- |
| **Infections during pregnancy? (yes/no)**  If yes, specify type of infection, trimester, and treatment  Influenza  Chest infection  Urinary tract infection  Hepatitis B  Hepatitis C  HIV  Others: please specify |
| **Pre-pregnancy height and weight (to calculate BMI)** |
| **Medication use during pregnancy (list medications used)** |
| **Smoking during pregnancy (number of cigarettes smoked per day)** |
| **Alcohol consumption (number of standard drinks consumed per week)** |
| **Socioeconomic status (postcode at time of pregnancy)** |
| **Pregnancy Complication Scale^a^:**  1, Optimal: No complications. Generally free of any discomfort.  2, Normal: No complications. Occasional periods of discomfort, no physical restrictions.  3, Mild: Mild complications that require an altered complications life-style but no medical interventions other than increased monitoring.  4, Moderate Moderate complications. One or more complications associated with some threats to mother's or fetus's well-being that typically require medical intervention.  5, Severe: Serious threat to health of mother or fetus, complications such as preeclampsia associated with the need for hospitalization or extended care. |
| **Complications present during pregnancy or delivery (yes/no)**  If yes, specify type of complication and trimester  High blood pressure  Proteinuria  Pre-eclampsia  Seizures  Hyperemesis gravidarum  Gestational diabetes  Placenta praevia  Spotting  Antepartum haemorrhage  Pregnancy-induced cholestasis  Threatened preterm labour  Fetal abnormalities or antenatal scan  Cervical shortening  Group B Streptococcal colonisation  Chorioamnionitis  Others: please specify |
| **Level of Stress Severity^a^:**  1, None: Notable events but not stressful.  2, Mild: Notable events associated with some increased stress, such as short-term changes in work schedule, brief periods of increased debt, or brief periods of heightened marital conflict.  3, Moderate: Events are associated with clear periods of increased stress, such as major change in job responsibilities, or illness of a family member.  4, Severe: Events are associated with severe stress typically resulting in a disruption of existing patterns of family life such as severe marital conflict with threat of separation, serious injury to a parent, or brief periods of unemployment.  5, Extreme: Stressful events associated with permanent or extended periods of time such as divorce, unremitting marital discord associated with physical abuse, unexpected loss of a family member, loss of residence, or life-threatening illness |

^a^The Pregnancy Complication Scale and the Level of Stress Severity have been previously used and published by Leckman et al. in a study exploring perinatal risk factors in Tourette’s syndrome.(Leckman et al. 1990)

**Supplementary Table 3. Capturing the child’s medical history and early life environmental exposures.** Section 3 of NDD-ECHO captures the child’s medical history, including childhood infections and exposure to stress using binary, checkbox, and multiple-choice questions on REDCap.

| **Height and weight (to calculate body mass index)** |
| --- |
| **Category: Autoimmune conditions**  Hashimoto's thyroiditis  Graves' disease  Thyroid disease not otherwise specified  Type 1 diabetes  Vitiligo  Alopecia  Psoriasis  Celiac disease  Crohn's disease  Ulcerative colitis  Systemic lupus erythematosus  Lupus-like syndrome  Rheumatoid arthritis  Uveitis  Multiple sclerosis  Pernicious anemia  Autoimmune hepatitis  Polymyalgia rheumatica  Others: please specify |
| **Category: Asthma and allergic-type conditions**  Asthma  Allergy (e.g. grass, pollen, food allergies, medication allergies)  Hayfever  Eczema  Other inflammatory conditions: please specify |
| **Childhood infection screener (Figure 1C)^a^**  Question: Has your child experienced any of the following infections or required medical attention for infections in the FIRST 5 YEARS or LAST 12 MONTHS of life?  Likert scale to assess frequency of infections: 0, Never; 1, Occasional (< 1 per year); 2, Sometimes (1-3 per year); 3, Often (4-6 per year); 4, Almost always (so frequent, hard to count)  Clear runny nose  Urinary tract infection  Throat infection/tonsilitis  Ear infection with pain or pus  Sinus infection  Pneumonia  Mouth ulcers  Skin infection (impetigo)  Meningitis or other serious infection (bone, joint, blood)  GP visit for infection  Antibiotic courses  Emergency department visit for infection  Hospitalisation for infection  Other significant infection |
| **Stressful life event with stressful reaction (yes/no)** |

^a^We created a purpose-built infection screening tool (see Figure 1C) which captures common viral and bacterial infections in childhood.

Leckman, J. F., E. S. Dolnansky, M. T. Hardin, M. Clubb, J. T. Walkup, J. Stevenson, and D. L. Pauls. 1990. 'Perinatal factors in the expression of Tourette's syndrome: an exploratory study', *Journal of the American Academy of Child and Adolescent Psychiatry*, 29: 220-6.
